# Simulated and measured piezoelectric energy harvesting of dynamic load in tires

**DOI:** 10.1016/j.heliyon.2024.e29043

**Published:** 2024-04-02

**Authors:** Henrik Staaf, Simon Matsson, Sobhan Sepheri, Elof Köhler, Kaies Daoud, Fredrik Ahrentorp, Christian Jonasson, Peter Folkow, Leena Ryynänen, Mika Penttila, Cristina Rusu

**Affiliations:** a RISE Research Institutes of Sweden, Smart Hardware Dept, Gothenburg, Sweden; b Breas AB – Sweden, Mölnlycke, Sweden; c Chalmers University of Technology, Division Dynamics, Gothenburg, Sweden; d Nokian Tyres Plc, Nokia, Finland

**Keywords:** Zero energy devices, Energy harvesting, Piezoelectricity, PVDF

## Abstract

From 2007 in US and from 2022 in EU it is mandatory to use TPMS monitoring in new cars. Sensors mounted in tires require a continuous power supply, which currently only is from batteries. Piezoelectric energy harvesting is a promising technology to harvest energy from tire movement and deformation to prolong usage of batteries and even avoid them inside tires.

This study presents a simpler method to simultaneous model the tire deformation and piezoelectric harvester performance by using a new simulation approach - dynamic bending zone. For this, angular and initial velocities were used for rolling motion, while angled polarization was introduced in the model for the piezoelectric material to generate correct voltage from tire deformation. We combined this numerical simulation in COMSOL Multiphysics with real-life measurements of electrical output of a piezoelectric energy harvester that was mounted onto a tire. This modelling approach allowed for 10 times decrease in simulation time as well as simpler investigation of systems parameters influencing the output power. By using experimental data, the simulation could be fine-tuned for material properties and for easier extrapolation of tire deformation with output harvested energy from simulations done at low velocity to the high velocity experimental data.

## 1 Introduction

The ever-increasing energy requirements pose one of the greatest technological challenges of our time. With an annual increase of around 1–2% in human energy use, the majority of energy comes from fossil fuels [1], which negatively impacts the environment. To address this challenge and as digitalization increases in many applications, alternative green sources of energy are emerging. One of the technologies that can aid in using less energy is energy harvesting, which converts ambient energy into electrical energy to power low-power sensor systems [[2], [3], [4], [5]]. This approach towards zero energy devices makes micro devices energy autonomous and can be placed in hard-to-reach locations, thereby positively impacting system installation and maintenance costs and time. It also helps in reducing the environmental impact by minimizing raw materials required for cable manufacturing and reducing the number of batteries thrown away [6]. The use of energy harvesting for powering sensing devices in tires is one such application that can enhance safety driving and automatic drive control [[7], [8], [9], [10]]. Due to regulations, from 2007 in US [11] and from 2022 in EU [12], it is mandatory to use tire pressure monitoring systems (TPMS) in new cars. All TPMS utilizes today battery as energy source, which is a burden to environment. To avoid using a battery inside the tire, energy harvesting is a green power solution with promising results as energy can be harvested from tire compression, deformations and vibrations when driving [[13], [14], [15]]. The energy harvester placement can be at the rim, inner liner, or side of the tire, with preferences for inner liner nowadays.

Initially, the main type of harvesters was piezoelectric PZT-based cantilever beam for TPMS [16,17]. Different cantilever structures/geometries were tested and while they provided a certain amount of energy (few μW/cm^2^ at 50 km/h), they have two major drawbacks. Firstly, there is limited space between the cantilever's gap and tire's deflection, which limits the length of the PZT cantilever beam, its deflection height and thus energy output. Secondly, PZT is highly brittle limiting the applied strain, and the beam is prone break easily as tire moves and deforms. Esmaeeli et al. [18] tried to improve the strain-based piezoelectric harvesters by designing a Cymbal shape obtaining 95 μJ per revolution at 41 km/h and about 600 kg load with an efficiency of ≈5%. Another piezoelectric-based material is poly (vinylidene fluoride) (PVDF) that is flexible, enables large deformation making it also good candidate for tire applications. even it has lower piezoelectric constant than ceramic PZT [[19], [20], [21]]. Lee and Choi [22] used PVDF film obtaining 380 μJ per revolution at 60 km/h and 500 kg load, converting and using approximately 9.7% of the available energy.

To design kinetic energy harvester, various software and methodologies are utilized, such as Finite element method (e.g. COMSOL, Ansys) and analytical equation (e.g., Matlab). Each harvester requires its specific simulation, depending on the application's requirements and specifications. Usually, the piezoelectric cantilever/beam harvester is subjected to base excitation arising from the radial deformation of the tire and centripetal acceleration due to the tire rotation [17] and from tire strain [18].

Simulations for tire deformation and energy harvester voltage output can be done analytically [8,17,18,23] and numerically. For example K. Anil [24] uses Matlab/Simulink software for modelling the design of piezoelectric ceramic placement at the tire-rim interface and tire deformation is introduced as a variable force; M. M. Behera [25] modelled the PZT energy harvester in MATLAB Simscape and simulated with tire using COMSOL Multiphysics 5.0 for output voltage. However, all these simulations are done by considering a static model of the deformation.

In this work, we present a new modelling procedure to simulate the combination of tire deformation and its influence simultaneously and dynamically on the attached piezoelectric harvester - dynamic bending zone – only the tire's bending zone (when in contact with the ground surface) is moving over the tire and harvester instead of the whole tire is rolling. This allows for much shorter simulation time, a more realistic simulation and modelling of deformation and its influence on harvester's deformation, simpler investigation of systems parameters influencing the output power (e.g. harvester geometry, type of piezoelectric material, tire pressure, car weight) and at the same time easier corelation with and improvement from experimental results. The experimental data allows for fine tuning of simulation in relation with material properties (e.g. Youngs modulus for tire and piezoelectric material). Thus, via experimental data at low velocities, it is easier in the simulation to extrapolate the tire deformation and harvested energy done at low velocity to high velocity. This easier method allows also for various shapes of the harvester to be simulated and benchmarked against experiments. None of the above capabilities can be easily done by using the existing state-of-the-art methods.

## 2 Modelling and simulations – tire and energy harvester

**Fig. 1:**
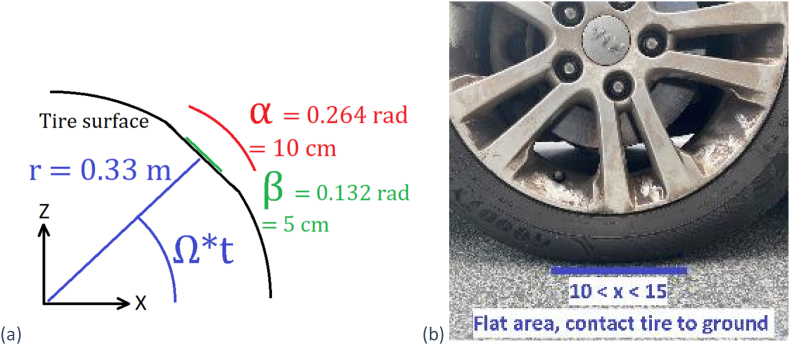
(a) Tire ground deflection circle arc in radians where ‘α’ and ‘β’ represent the contact length and energy harvester length, respectively, (b) Estimation of the flat area during deformation.

A simple model of a tire was first simulated in COMSOL Multiphysics 5.0 to determine its mechanical properties. The model consisted of a structural steel cylinder surrounded by a thin layer of rubber with Young's modulus of 50 MPa and Poisson's ratio of 0.3, resting entirely on the ground. To simulate a real tire and avoid the flat roll area between the ground and the tire, two angles ‘β’ and ‘Ω’ was introduced between the roll area and the side wall (Fig. 1(a)).

At first, the tire was simulated in a stationary state with a body load of 500 kg distributed over an area approximating the contact patch of the tire by using cosine and sine factors to apply the load in the correct direction.

The contact area of the tire with the ground was approximated to be 10 cm long (Fig. 1 (b)), the vertical deformation is 0.36 cm–0.8 cm, the entire circumference of the tire was 2.39 m, which corresponds to 2π radians. This allowed the 10 cm length to be converted to an angle α ≈ 0.264 radians (Fig. 1(a)). The load was applied in the direction perpendicular to the centre of the contact patch defined by $\vec{n}=-\left(r\cdot \cos\left(\Omega t\right),0,r\cdot \cos\left(\Omega t\right)\right)$, where *r* is the radius to the outer edge, Ω is the angular velocity of the tire and *t* is the time. In the interest of simplicity, the carload was applied to only one quarter of the tire. To ensure a non-radial load, the angle of the load was defined by the normal to the middle of the surface defined by α. When simulating the curved tire, the angular velocity was calculated to match the velocities that were going to be used during the experimental phase.

**Fig. 2:**
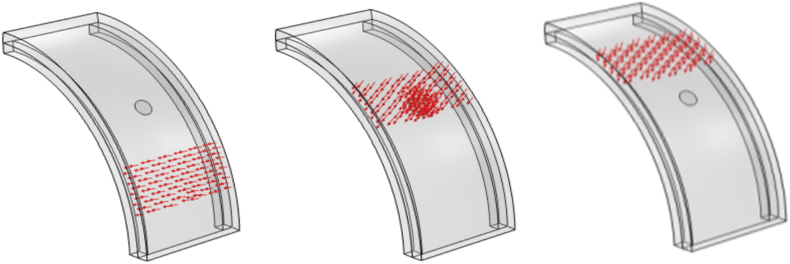
Comsol simulation of tire where the deformation load is moved over the surface of the tire.

To simulate rolling motion, angular velocity and initial velocities along and across the ground were used. However, these simulations were time-consuming and required numerous iterations to avoid errors. Thus, the model was changed such that the force load (simulating ground) was made to move over the tire instead of moving the tire itself (Fig. 2).

To model the pressure inside the tire, a cavity in the middle of the tire is added to represent the area where air is inflated in real tires. A standard pressure value is chosen of 2 bar, which was modelled radially outward in a quarter of the tire where the carload was applied.

At the edges of the simulated quarter-tire a boundary condition was applied to prevent azimuthal deformations while still allowing radial deformations. On the inner edges of the tire wall, which rest on the rim a similar boundary condition was applied. However, in this case it fixes the position in all directions.

**Fig. 3:**
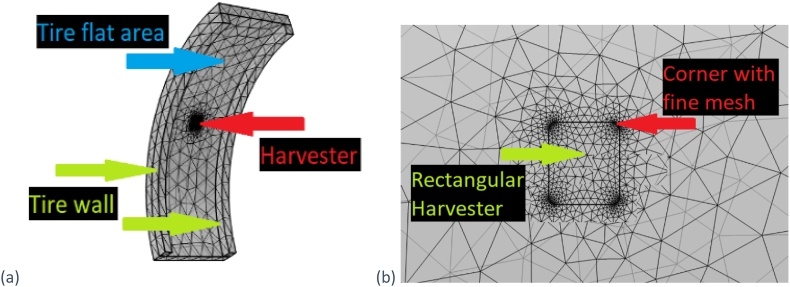
(a) Meshed tire geometry (b) Needed finer mesh around the harvester (at the corners compared to straight section).

The flexible PVDF-TrFe piezoelectric harvester component was added as a 0.01 cm thick layer, 5 cm long (β = 0.132 radians, Fig. 1(a)) and 2 cm wide. The entire meshed geometry of both the tire and a rectangular harvester can be seen in Fig. 3(a). Rounded corners for square/rectangular harvesters had to be introduced (Fig. 3(b)) to avoid stress concentrations as 90° corners create, that resulted in easier meshing and faster solving time.

The ground was defined as the inner edge of the piezoelectric PVDF-TrFe harvester, and to simulated open circuit voltage, a load resistor of 10 MΩ in parallel to the harvester was added to the model.

The PVDF-TrFe harvester is polarised for d33 and its material's properties are approximated from literature with d33 value of −33.8 pC/N. The material data for rubber was estimated from literature with Young's modulus of 0.19 GPa (as the tire rubber is a mixed combination of different types of materials).

An angled polarization was introduced in the model for the piezoelectric material – oriented inward toward the centre of the tire as the d33 direction – to generate accurate voltage from deformation.

The dependence of the harvester's geometrical shape on the output voltage was also investigated, rectangular and circular shapes by maintaining the same area. However, the area was varied allowing for an investigation of area dependency rather than solely length or thickness dependency.

## 3 Simulation result

**Fig. 4:**
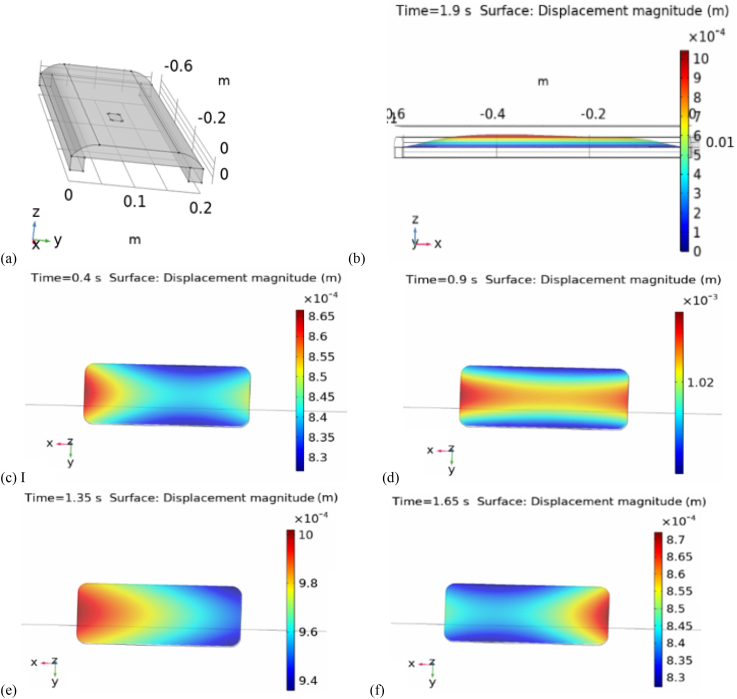
Comsol tire simulation (a) meshing of area under deformation, (b) cross-section of deformation showing the vertical displacement due to the double deflection (‘contact’ and ‘leaving’), (c)–(f) top view deformation with time. Tire rubber: Young's modulus = 0.19 GPa, length = 0.6 m, width = 0.2 m, thickness = 2 cm; PVDF-TrFE harvester length = 5 cm, and width = 2 cm.

The simulations of rotating tire in contact with a physical ground took long time even for very low velocities (e.g. 0.5 m/s) but changing the methodology by having the load moving over the tire, higher velocities could be simulated within reasonable simulation times. These results could be successfully correlated with experiments at various velocities. The simulated tire deformation and displacement behaviour acting on PVDF-TrFe harvester are presented in Fig. 4(a–f). By collecting all displacements from the force load moving over the harvester area, the value of total displacement was obtained. Curve fitting was performed on these values to obtain equations that described the displacement with best curve fit as shown in equation (1); displacement ‘d’ [m] and the angle ‘Θ’ [radian] over which the load was applied:

(Eq. 1) d = −0.3407 · Θ^4^ + 0.1815 · Θ^3^ – 0.02646 · Θ^2^ + 0.0006164 · Θ – 5.078 · 10^−6^

In Fig. 4(a–f), it can be seen the behaviour of the deformation on and along the tire and thus on the harvester during time evolution of tire in contact with the ground.

The output voltages from COMSOL Multiphysics is direct scalable to the weight load over the surface; an increase in carload from 200 kg to 500 kg resulted in a voltage output increase by a factor of 2.5. However, increasing the force load's velocity over the tire surface resulted in a non-linear increase in the voltage generated by the PVDF harvesters, following a 2nd order equation up to a fixed value where it remained constant (not shown here).

**Fig. 5:**
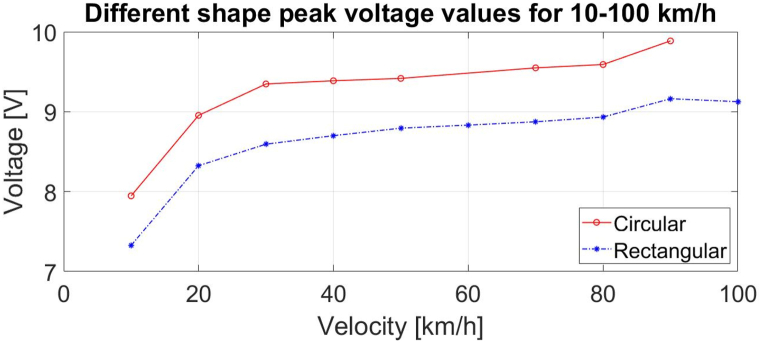
Simulated peak open-circuit voltage values at different velocities for different geometrical shapes - circular harvester has higher output compared to rectangular. The total area of each shape is the same at 4.cm^2^.

**Fig. 6:**
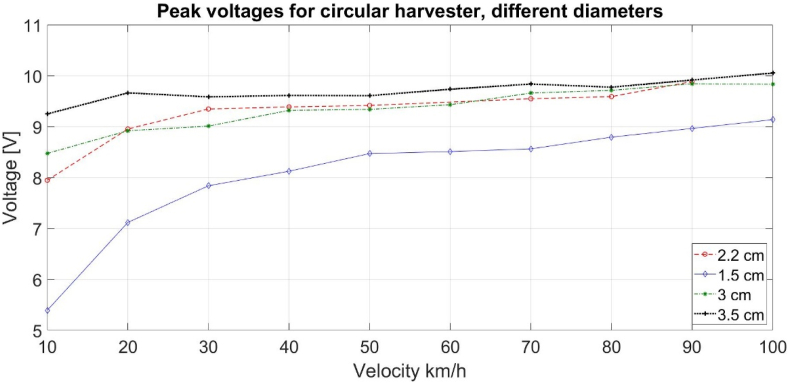
Simulated open-circuit peak voltage at different velocities for different circular harvester sizes.

Rectangular and circular geometrical shapes were analysed using both COMSOL Multiphysics simulations and real-life experiments. The simulations were performed keeping constant the harvester polarization angle and the harvester footprint areas. The resulting voltages have a similar behaviour as those from experimental results with higher output open-circuit voltage for the circular harvester, Fig. 5. Various circular areas were simulated showing that no voltage increase happens after a certain diameter, Fig. 6.

## 4 Measurement method

**Fig. 7:**
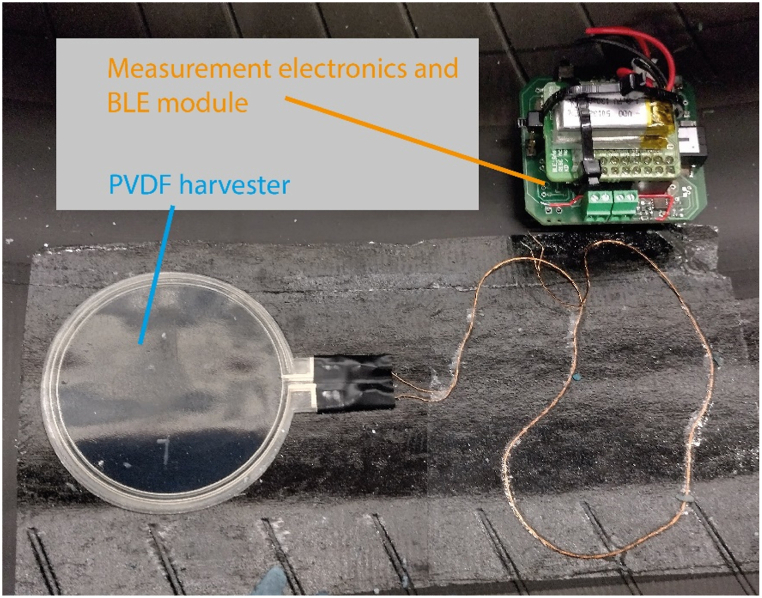
PVDF-TrFe harvester film attached to the inside of tire with double sided tape and connected to the measurement electronics and BLE module.

The PVDF-TrFe harvesters were fixed onto the tire using double-sided tape and connected to measurement electronics module that also incorporates a Bluetooth Low Energy (BLE) unit for sending the data wirelessly during tire measurements, Fig. 7. The measurement electronics have a 470 μF capacitor and a 10 MOhm matching resistance and it measures various electrical parameters for harvester characterization, charging slope, open-circuit voltage, voltage output. The saturated voltage of the capacitor represented the maximum voltage response of the PVDF harvesters as a function of speed.

**Fig. 8:**
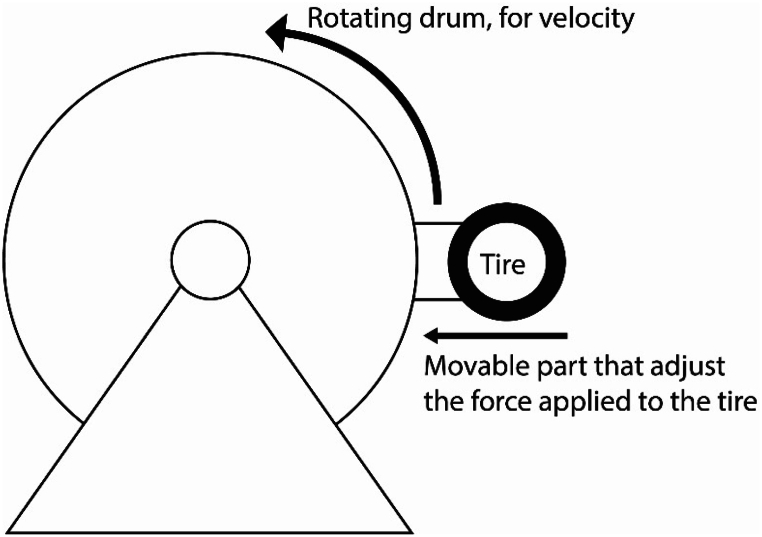
Schematic of a typical drum setup for tire testing.

Testing of energy harvester on the tire is done in the specialised lab at Nokian Tyres PLC with conditions as close as possible to real-life environmental situation – tire pressure, car load, velocities, Fig. 8.

## 5 Results and discussion

**Fig. 9:**
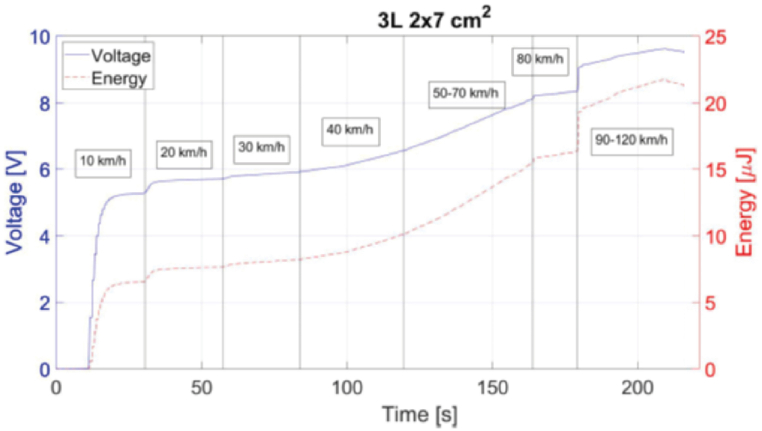
Measured PVDF harvester voltage and energy for three-layer 2 × 7 cm^2^ harvester with 2 bar tire pressure, 200 kg carload and velocities from 10 to 120 km/h.

**Fig. 10:**
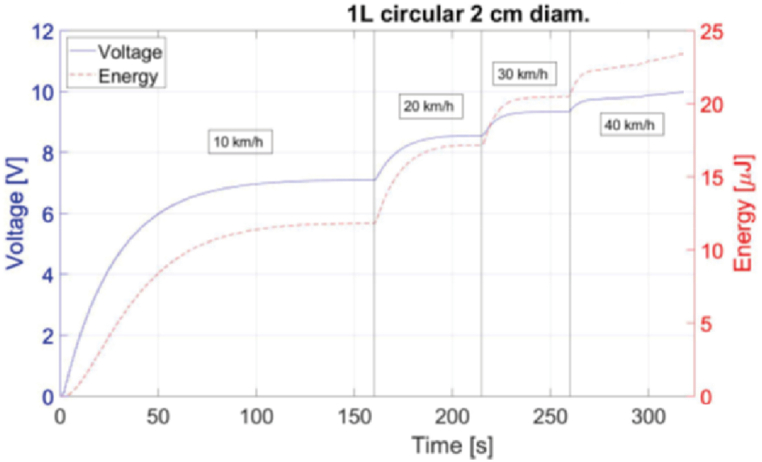
Measured harvester voltage and energy for single layer 2 cm diameter circular harvester with 2 bar tire pressure, 200 kg carload and velocities from 10 to 40 km/h.

Experimental tests were performed at Nokian Tyres PLC test facility to characterize and select the best energy harvester regarding voltage and energy output. Measurements were conducted with carload of 200 kg and 2 Bar pressure. Various designs were tested (e.g. one-layer or multiple layers, square/rectangular/circular). A typical behaviour, charging and saturated voltages, and energy for a three-layer 2 × 7 cm^2^ harvester with varying velocities ranging, 10 km/h to 120 km/h is shown in Fig. 9. The maximum accumulated energy was approximately 22 μJ. Another example of harvester design and its performance is one-layer circular harvester with energy and saturated voltages at different velocities (-0 km/h to 40 km/h) is shown in (Fig. 10) - 7.1 V, 8.3 V, 9.1 V, and 10 V, respectively, with a maximum accumulated energy of 23 μJ.

It is seen in these two figures that for lower velocities the voltage reaches a saturation value/plateau much faster than for higher velocities. This can be due to variation of self-leakage in the capacitor.

From the experiments, the charge rate for the 1 cm^2^ harvester was 1.65 nC/s, while for the 3-layer harvester was 69.3 nC/s, both at 20 km/h. The charge rate nC/(s*cm^2^) is the same for both rectangular and square harvesters regardless of their dimensions and number of layers, as presented in Table 1. The charge rate per area for the circular shape is nearly 10 times higher compared to the rectangular one, most probably because a bigger area is utilized as active harvesting (and this is under further investigation).

**Table 1:** Comparison of the measured voltage and charge rate per area for 20 km/h.

Geometry harvester	Area **[cm**^**2**^**]**	Sat. V	V/cm^2^	Charge rate [nC/(s*cm^**2**^**)]**
Three-layer 2 × 7 cm^2^ rectangle	42	5.7	0.136	1.65
1 cm^2^ square	1	N/a	N/a	1.65
2 cm diameter circle	*π*	8.5	2.71	16.8

**Fig. 11:**
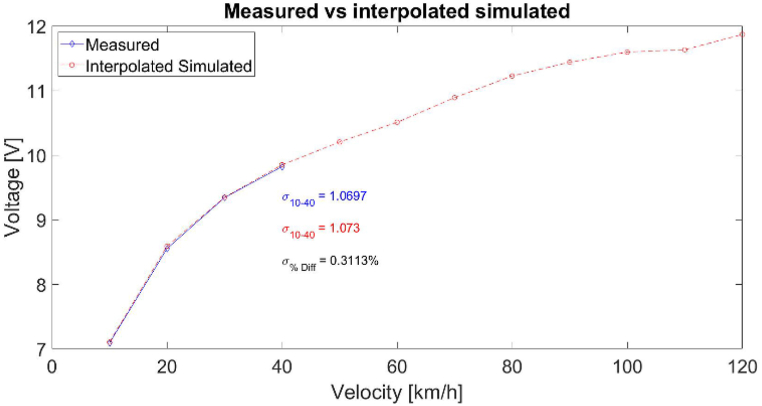
Graph showing the measured voltage for 10–40 km/h and the simulated data multiplied with the factor equation for 10–120 km/h.

It can be seen that there is a small discrepancy between the simulated voltages and the ones measured in output value, Fig. 5 vs Fig. 9, Fig. 10. This difference in output voltage is most likely due to various factors such as the adhesive used for harvester mounting onto the tire that was not simulated and, we could see that it did not keep the harvester on place (there was shift/slide in the position), the actual values for tire's Young's modulus and Poisson ratio due its complex materials combination. To improve the simulation for these issues, we utilized simulations and measurements data for the same harvester geometry and velocities to calculate the factors between the two data sets and derived the best equation fit. Thus, we obtained a very good match for the range 10–40 km/h allowing to estimate the voltages' values for higher speeds than simulated and/or measured, as can be seen in Fig. 11. Moreover, this type simulation and experimental equation fit procedure can be done once for low velocities measurements and then just simulate for higher velocities for PVDF energy harvesters (without the need for experiments at higher velocities).

## 6 Conclusion

The paper describes an easier model and simulation strategy such that both the tire deformation and output energy from flexible piezoelectric harvester mounted on the inner tire can be simultaneously simulated. In this ‘dynamic bending zone‘ approach, the force load moves over the tire and harvester instead of the tire rolling facilitating converging simulations results within realistic time. The model is flexible, allowing for easy parameters modification for tire and harvester geometry, material, and conditions. The simulated model shows very good relation to the experimental results. Even if the first simulations are done for low tire velocity and with no exact material values, the feedback from experiments permit the improvement of the simulation model. By using dynamic bending zone, the simulations could be done with high confidence for various harvester designs and higher velocities without the need for experimental data.

The simulations and measurements have given insights into the characterization and behaviour of PVDF-based harvester for tire application. It shows that the geometrical shape has a large impact on the output with circular harvester showing higher saturated voltages and charge rates compared to rectangular and square harvester.

The measured output energy is in principle enough to power some type of sensors already in used for tire behaviour and its environment characterization. Overall, the study provides valuable insights into the design and optimization of piezoelectric harvesters for energy harvesting in tires.

## CRediT authorship contribution statement

**Henrik Staaf:** Writing – review & editing, Writing – original draft, Validation, Supervision, Software, Conceptualization. **Simon Matsson:** Writing – review & editing, Formal analysis, Conceptualization. **Sobhan Sepheri:** Writing – review & editing. **Elof Köhler:** Writing – review & editing. **Kaies Daoud:** Software. **Fredrik Ahrentorp:** Software. **Christian Jonasson:** Supervision, Software, Conceptualization. **Peter Folkow:** Writing – review & editing, Supervision, Investigation. **Leena Ryynänen:** Resources. **Mika Penttila:** Resources. **Cristina Rusu:** Writing – review & editing, Writing – original draft, Supervision, Project administration, Investigation.

## Declaration of competing interest

The authors declare the following financial interests/personal relationships which may be considered as potential competing interests Cristina Rusu reports financial support was provided by 10.13039/501100000780European Commission. If there are other authors, they declare that they have no known competing financial interests or personal relationships that could have appeared to influence the work reported in this paper.
